# Development of a Multi-Material Stereolithography 3D Printing Device

**DOI:** 10.3390/mi11050532

**Published:** 2020-05-22

**Authors:** Bilal Khatri, Marco Frey, Ahmed Raouf-Fahmy, Marc-Vincent Scharla, Thomas Hanemann

**Affiliations:** 1 Department of Microsystems Engineering, University of Freiburg, Georges-Koehler-Allee 102, D-79110 Freiburg, Germany; bilal.khatri@inatech.uni-freiburg.de (B.K.); marco.frey@imtek.de (M.F.); ahmed.fahmy@tum.de (A.R.-F.); marc.scharla@web.de (M.-V.S.); 2 Karlsruhe Institute of Technology, Institute for Applied Materials, Hermann-von-Helmholtz-Platz 1, D-76344 Eggenstein-Leopoldshafen, Germany

**Keywords:** additive manufacturing, stereolithography, photopolymers, multi-material 3D printing

## Abstract

Additive manufacturing, or nowadays more popularly entitled as 3D printing, enables a fast realization of polymer, metal, ceramic or composite devices, which often cannot be fabricated with conventional methods. One critical issue for a continuation of this success story is the generation of multi-material devices. Whilst in fused filament fabrication or 3D InkJet printing, commercial solutions have been realized, in stereolithography only very few attempts have been seen. In this work, a comprehensive approach, covering the construction, material development, software control and multi-material printing is presented for the fabrication of structural details in the micrometer range. The work concludes with a critical evaluation and possible improvements.

## 1. Introduction

Starting with the brilliant invention described in “Apparatus for production of three-dimensional objects by stereolithography” in 1986 by Charles W. Hull [1], the development of different 3D printing techniques has happened at a brisk pace. This has made possible the realization of polymer, ceramic and metallic parts with geometrical features previously inconceivable due to the topological limitations of traditional fabrication methods, such as mechanical machining and injection molding. In addition to stereolithography (SLA), a wide variety of methods are now established under the umbrella of additive manufacturing, such as fused filament fabrication (FFF), 3D InkJet printing (also known as PolyJet^®^) or powder-based printing (binder jetting, laser or electron sintering or melting (SLS, SLM, EBM)). This speedy advancement of additive manufacturing technologies, as well as that of 3D printable materials, such as curable resins for SLA or PolyJet^®^, thermoplastics for FFF and fine metal powders for SLM or EBM, have led to a technological readiness for reliable rapid prototyping and, in some cases, even small-scale production [2,3,4,5,6]. However, and in contrast to established macroscale fabrication methods, there are still some open questions and fields, which require solving and answering for further development of this emerging field [7]. In addition to design and shape optimization, development of printing methodologies, digital material development, error control and modeling issues, a key challenge for the further dissemination of 3D printing into the industry is that of materials, in particular multi-material printing. In FFF, the printing of functional polymer matrix composites with ceramic and metal fillers is established [8,9,10], even for the realization of dense ceramic and metal parts, in an approach analogous to powder injection molding [11,12,13]. A first two-component FFF, combining the printing of zirconia and stainless steel filaments and subsequent thermal post-processing was reported in 2019 [14]. Multi-material printing for FFF was first reported in 2002 [15], dealing with the modeling of the printing strategy optimization. Since then, the combination of electroplating and FFF has been investigated by Matsuzaki and recently by Ambrosi et al. [16,17]; a comprehensive review can be found in [18]. In the case of SLA, the composite printing of ceramic-filled resins, curing and thermal post-processing has been commercialized [19,20].

On the multi-material SLA side, an early work dealing with multi-material stereolithography was published in 2006 by Inamdar et al. [21] using a modified commercial SLA machine (3D Systems 250/50) and the implementation of a rotating vat carousel system. The vats had a volume around 9 L and a resin pump filling/leveling system. The resin polymerization was induced by a 355 nm solid state laser. The authors claimed a precision of the z-stage of ± 20 µm and a repeatability of ± 1 µm controlling the build layer thickness. Unfortunately, the printed test samples were not further characterized geometrically [21]. A more detailed description was given in [22], also introducing a rotary platform stage. The same research group developed an alternative system using only one vat; the resins can be exchanged using a syringe pump after manual cleaning and rinsing [23]. In the latter case, a layer thickness around 21 µm was possible. However, details about the x, y-resolutions were not discussed [23]. A comprehensive review summarizes both developments [24]. The authors extended the portfolio of usable curable materials to, e.g., polyethylenglycol (PEG) based hydrogels or conductive layers for electronic circuit fabrication [24]. In a hybrid approach, combining stereolithography with aerosol jet printing, a continuous mixing ratio of two different resins and subsequent UV curing was realized [25]. Roach et al. [26] implemented a combination of different 3D printing methods and related materials in 2019. Another early multi-material SLA system operating with two vats was presented by Zhou et al., in 2011, applying a digital micromirror device (DMD) serving as a digital light processing (DLP) modulator with a resolution of 1024 × 768 pixels [27]. The achieved structural details were in the sub-millimeter range. Two different polyethylene diacrylate-based hydrogels have been used for the printing of micro-channels, including diffusion barriers also using a DMD projector and intermediate vat change. Using this system, micro-channels with dimensions around 200 µm could be realized [28]. Another hybrid approach, combining DLP–SLA with a drop-on-demand (DoD) Inkjet printing system, was presented by Muguruza et al. in 2017 [29]. As an upcoming trend, the combinations of different additive manufacturing techniques for the realization of multi-materials combining individual strengths is gaining more prominence [30].

For the additive manufacturing of polymeric multi-material components, three different methods should be considered: Extrusion-based techniques like FFF and the ones making use of curable resins, like 3D InkJet (PolyJet^®^) and SLA. Modern FFF two-component printers are commercially available below €10,000 and show resolutions of 20 µm in z- and 100 µm in x-, y-directions on paper. Realistically achievable values are at least double the given values for commercial thermoplastics and may not be repeatable for each printing position or shape. The realization of structural features below 100 µm is hindered by these issues. The use of functional composites, e.g., with dielectric or magnetic properties for the realization of ceramic or metallic parts, requires additional melt-compounding steps such as mixing–kneading and filament extrusion [8,9,11,31]. Due to the round filament shape and its deposition in a layer-by-layer manner, poor inter-layer adhesion within one component is one of the major drawbacks of single-material [31] and two-component FFF printing [32]. Commercial multi-component 3D InkJet printers are available at prices upwards of €30,000, enabling geometric resolutions better than 50 µm in x-, y- and z-directions. Unfortunately, the user is restricted to a set of fixed materials and closed software, both offered by the machine vendor. Material development for DoD printing is difficult due to its piezo-driven inkjet print-head, because the ink viscosity must be lower than 20 mPas at the printing temperature and all dispersed particles for the realization of functional composites must be smaller than 1 µm in order to avoid print-head nozzle clogging [33,34]. Individual layers cannot be detected after printing and post-curing, resulting in good mechanical properties. Currently, SLA is the best compromise, considering different aspects such as the material portfolio, potential for new material development, multi-material printing, geometric resolution, machine investment and consumable materials. The only disadvantage is the limitation of photocurable resins as the build material due its operating principle.

The aim of this work is the development of a new, versatile, multi-material micro-stereolithography 3D printing device (MMSL), developed using low-cost commercial equipment and components, with the usage of, at present, up to three individual curable resins without intermediate material exchange, enabling a more flexible material combination. The functionality of the new MMSL device will be shown by the combination of a variety of curable acrylates with different fluorescence markers.

## 2. Materials and Methods

**Figure 1:**
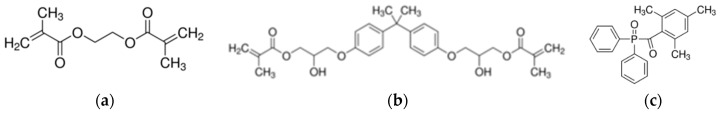
Photocurable resin composition: (**a**) EGDMA; (**b**) BAEDA; (**c**) TPO.

Following earlier investigations on the photocuring behavior of diacrylates, ethylene glycol dimethacrylate (EGDMA, Merck, Darmstadt, Germany, Figure 1a) and bisphenol A glycerolate dimethacrylate (BAEDA, Merck, Darmstadt, Germany, Figure 1b) were selected as suitable monomers for approval as a curable base material for MMSL development [35,36]. Diphenyl(2,4,6-trimethylbenzoyl)phosphine oxide (TPO, TCI, Frankfurt, Germany) was selected as a photoinitiator due to its good light absorption in the range between 350 and 430 nm and with ratios between 0.1 and 3 wt.% (Figure 1c). Different fluorescent dyes were used for better visualization of the printed MMSL structures. Initial experiments used an europium-based organic complex ((Tris (1,3-diphenyl-1,3-propanedionato) (1,10-phenanthroline)europium(III) (TCI, Frankfurt, Germany), which is responsive to near-UV radiation. Subsequent experiments used two commercially available fluorescent dyes, the perylene-based Lumogen F305 (red) and the naphthalimide-based V570 (violet) (BASF, Ludwigshafen, Germany), both at a concentration of 0.05 wt.% in the resin matrix. All resin mixtures were prepared using the Ultra-Turrax T-10 disperser (IKA, Staufen, Germany) by mixing at 15,000–18,000 rpm for 3–5 min under ambient conditions.

The viscosities of the EGDMA-BAEDA mixtures were measured using a cone and plate rheometer (CVO 50, Bohlin/Malvern Instruments, Herrenberg, Germany) at shear rates between 10 and 500 1/s and in the temperature range between 20–50 °C. The resin suitability for SLA 3D printing was evaluated using the commercially available B9Creator SLA printer (B9Creations LLC, Rapid City, IL, USA), which uses DLP technology and is capable of voxel resolutions down to 30 µm. A post-print flood exposure was performed on all samples using the 600 mW/cm^2^ Hönle LED-Spot-100 UV lamp (Dr. Hönle AG, Gräfelfing, Germany) at 365 nm for 120 s. Tensile tests (five samples) according to ASTM D-638 were performed applying a Zwick/Roell Z010 universal testing machine (Zwick/Roell, Ulm, Germany) under ambient conditions (2.5 kN load cell, pull speeds of 2 mm/min and 5 mm/min). In the case of the systematic investigation of BE-5050, doped with the Lumogen chromophors, five samples were investigated for each selected layer polymerization time. The size of the specimen was reduced by a factor of 0.4 due to vat volume restrictions.

## 3. MMSL Printing Device

### 3.1. Design and Construction

Following the restrictions of earlier approaches dealing with multi-material SLA devices and potential consumer wish lists, the following set of requirements for the new device was defined:

- Simple and low-cost construction and setup.
- Integration of building blocks, like a DLP light source, from established commercial systems.
- No material exchange during the printing procedure.
- Realization of structural features below 100 µm with good reproducibility.
- Simple, adaptable control software.

**Figure 2:**
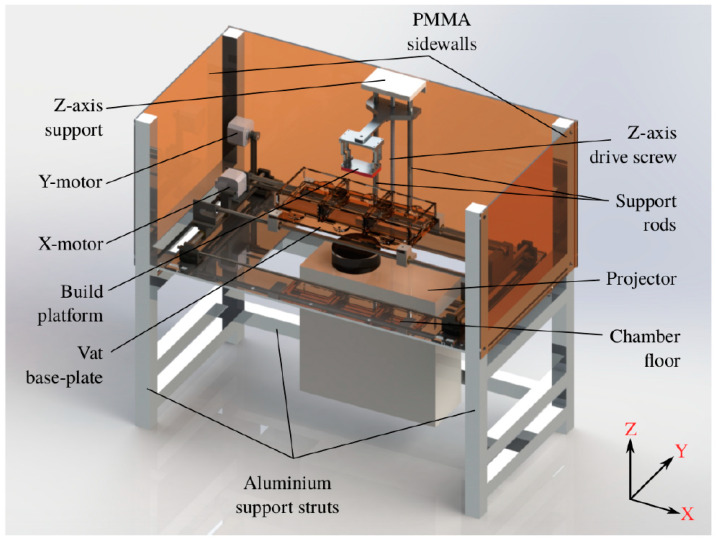
A SolidWorks^®^ -designed 3D model of the MMSL device.

Figure 2 shows the initial design of the MMSL device using SolidWorks^®^. The device’s housing was constructed from aluminum struts (Bosch Rexroth, Stuttgart, Germany), with a milled aluminum sheet forming the bottom of the printing, an illumination chamber with openings for the attached projector and mounting points for the linear stages. Transparent polymethylmethacrylate (PMMA) sheets, covered with Kapton^®^ foil, were used for all sidewalls, as well as for the front door of the chamber. An opaque yellow PMMA sheet was used as the chamber roof to reduce ambient light. An 80 mm fan helped to remove the heat generated by the projector out of the chamber. Individual stepper motors were applied for the three linear x-, y- and z-stages for the movement of the vats and the build-platform, with additional mechanical homing switches attached to each stage. These motors, stages and switches were reused from an out-of-action FFF printer (MakerBot 2X, MakerBot Industries, New York City, NY, USA) and modified for our purposes.

**Figure 3:**
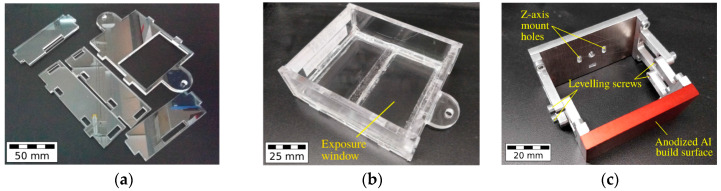
MMSL resin vat: (**a**) Laser cut PMMA sheets; (**b**) Assembled vat; (**c**) Build platform.

**Figure 4:**
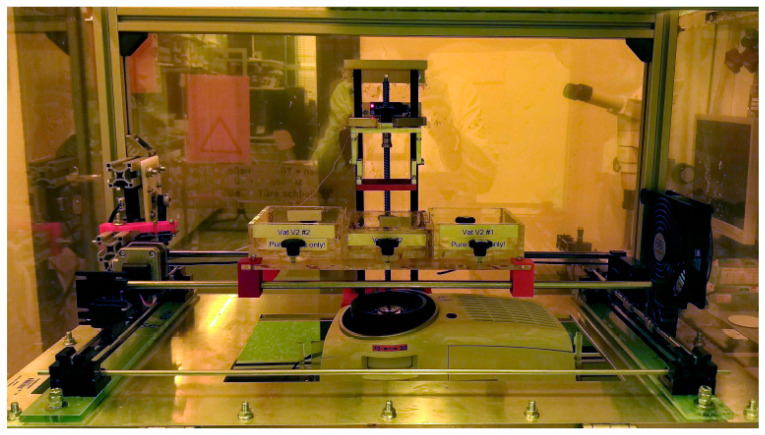
Ready to use MMSL device setup.

The laser cutting of PMMA sheets allowed for the prefabrication of the individual vat parts (Figure 3a), which were assembled by gluing to the final vat (Figure 3b). The vats were designed to have an inner area of 80 × 95 mm^2^ and were designed to work with small resin volumes of around 30 mL. They were equipped with a glass illumination window, covering around 50% of the vat’s inner surface area (Figure 3b). The vat window was varnished with a non-stick silicone layer (Sylgard^®^ 184 optical-grade, Dow Corning, Midland, MI, USA) to provide a stable, optically transparent, yet soft inner surface. For this, around 20 g of liquid Sylgard^®^ 184 was evacuated before being poured onto the vat window and cured at 60 °C for up to 4 h. All vats were fixed on a PMMA platform and placed below the aluminum build-platform with a build-surface of 64 × 36 mm^2^ (Figure 3c). The build-platform assembly was equipped with four tilt-adjustment points to help the build-surface to be calibrated parallel to the vat window. The build-platform was connected to the z-axis arm by a thumb-screw, which in turn was mounted on to a stepper-driven 280 mm long acme lead-screw with a 2 mm pitch. A modified version of the Vivitek D912HD DLP projector (Vivitek, Hoofddorp, The Netherlands), in its 30 μm pixel mode, and with a build area of 57.6 × 32.4 mm^2^, was used as an illumination source. Due to filters filtering UV-B and UV-C radiation, the exploitable wavelengths for photopolymerization are in the range between 375–405 nm. Figure 4 shows the mounted MMSL device after completion. The maximum size of printable parts is 57.6 × 32.4 × 50 mm^3^.

### 3.2. Device Control

As mentioned above, three NEMA-17 stepper motors, reused from a dismantled FFF printer, were connected to the printer stages and controlled by an Arduino UNO microcontroller mounted with a CNC shield overlay. The latter allows for up to four stepper motor drivers, three of which were connected to the stepper motors using Polulu stepper motor carriers (Polulu Corporation, Las Vegas, NV, USA), each equipped with the DRV8825 high current stepper driver. The printer design required three unique positions on the x-axis for vat selection and two on the y-axis for illumination and layer delamination, while simultaneously establishing and breaking the line-of-sight between the projector and build-platform. These motors worked in the 1/8th microstepping mode to enable smooth operation, in contrast, the z-stage was configured at 1/16th microstep enabling small layer thicknesses from 100 μm down to 10 μm. The Arduino was connected to a desktop PC via USB and controlled by LabVIEW software (National Instruments, Austin, TX, USA) applying the Arduino control interface, the same is valid for the projector using a serial RS-232 connection.

### 3.3. Software: GUI and Printing Programs

**Figure 5:**
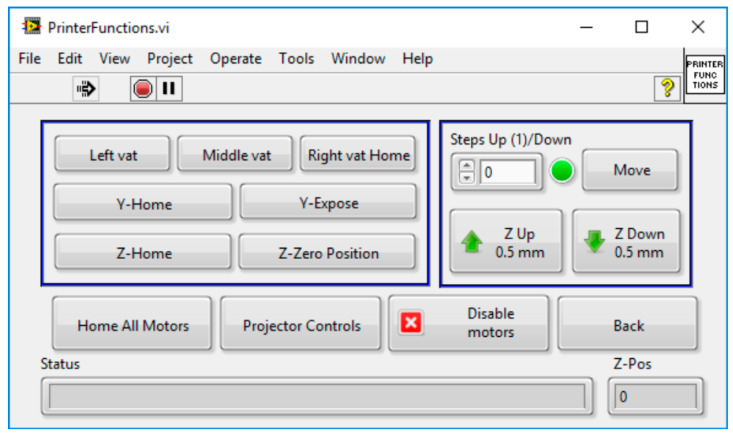
Printer functions screen with motor control.

The homemade MMSL control software allows the import of slice files in the established JPG, PNG or BMP formats. These slice files were generated using the B9Creator software, which was additionally used to position, scale and orient the 3D models. The MMSL software is capable of working directly with this slice data, thereby reducing the effort for additional GUI programming. All relevant print parameters, like illumination time, motor movement and delays before and after exposure could be adjusted in the software. The software was programmed in a top-down hierarchical manner using LabVIEW state machines. The main user interface called subroutines for calibration sequences, printer functionality tests or the printing programs, allowing the user to define individual process parameters, like moving a motor or switching the projector on or off. When started, the program first undergoes a self-test by establishing and verifying communication with the Arduino and the projector, after which subroutines to calibrate the build-platform and projector, as well as those to test individual motor functions, can be called. Figure 5 shows exemplarily the printer functions screen including motor control. The MMSL software includes two calibration programs, one each for the projector and the build-platform. Both programs follow a checklist-style interface to minimize unwanted motor movements. The projector calibration sequence first homes all three axes sequentially and switches on the projector to display a calibration grid. Coarse and fine focal adjustments can be manually done by positioning the two focus-rings on the projector. The sharpness of the calibration grid can then be viewed on different vats. The build-platform calibration program allows the build-platform to be adjusted parallel to the vat window.

The MMSL software has three sub-programs each for single-material and multi-material print-jobs. The first is used to select and adjust the print parameters, followed by a pre-print checklist and the print program displaying the status of the print-job being performed. Both print-setup programs first prompt the user to point to a folder with the slice files in JPG, PNG or BMP format and the appropriate print parameters. The pre-print checklist prompts the user to sequentially position the motors, switch-on the projector and fill the vats with resin. After the print-job has begun, no further intervention from the user is necessary. Multi-material prints involve the use of up to three vats during a single print job. The program requires a print recipe in the form of a CSV file, where the vat, the exposure time and the start- and end-layers for each print step are defined. To minimize the cross-contamination of different resins, a rinse step can also be integrated in the print recipe. In this case, typically the middle vat is filled with a suitable organic solvent, like isopropanol, which is compatible with the PMMA vat.

**Figure 6:**
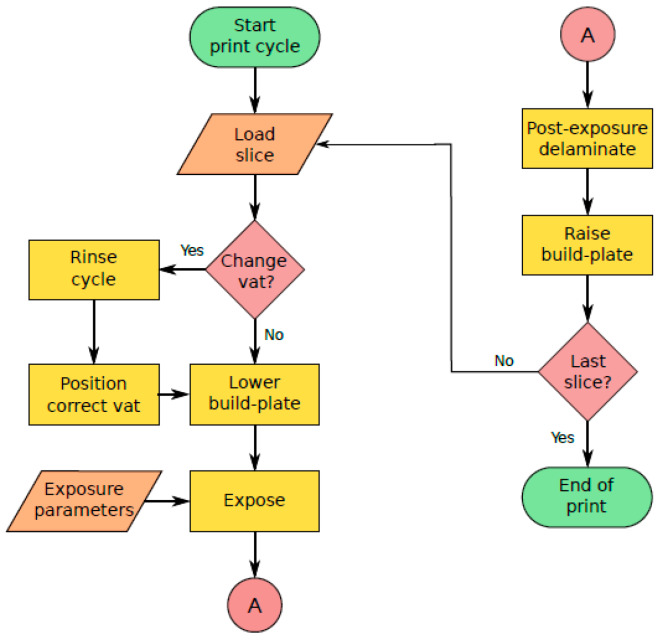
Print-cycle flowchart of the MMSL device.

The MMSL device works in print-cycles, where the completion of a print-cycle corresponds to the photocuring of a layer of a defined thickness. Figure 6 shows a flowchart describing a print-cycle for a multi-material print. The print-cycle begins with the lowering of the build-platform into the vat, to a depth equal to one layer-thickness (adjustable between 10 μm and 100 μm) less than that of the last layer. The program then waits for the resin to reflow in between the build-platform and the vat window. The current slice image is then exposed for a user-defined period, polymerizing the resin layer. For the first layer, a longer exposure time should be used to ensure the adhesion of the solidified material on the build-platform. After one illumination is finished, the vat-platform moves along the y-direction away from the vat window (axis orientations defined in Figure 2). This lateral movement step is important to protect the silicone on the vat window from damage, as a direct upward movement of the build-platform would result in an upward suction and possible delamination of the silicone from the vat’s body. After decoupling with the silicone, the build-platform can move upwards by a defined amount, followed by a y-stage movement to re-establish the line-of-sight between the build-platform, the vat window and the projector, enabling the next exposure cycle. In the case of multi-material prints, the print-cycle algorithm additionally checks if the previous layer was the last one for the current print step and can home the build-platform, moving it out of the way for a change of vats or a rinse cycle. After all layers are finished, the x-, y-, and z-motors are sequentially homed, the projector is turned off and the build-platform can be manually detached; finally the printed structure can then be manually removed from the build-platform and post-processed if necessary.

## 4. Resin Development and Characterization

**Figure 7:**
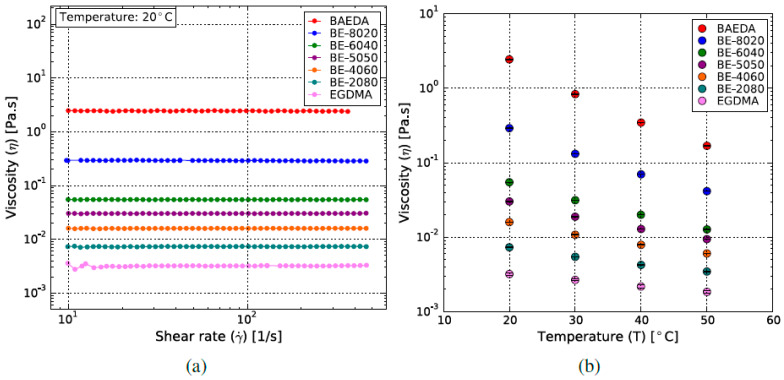
Viscosities of all investigated resin mixtures as function of (**a**) shear rate and (**b**) temperature.

Previous work has shown that resins developed in our research group are compatible with the B9Creator and its illumination source [37]. Building up on this, experiments showed that a new base resin comprising BAEDA and EGDMA as comonomers, and in combination with TPO as a photoinitiator, had the best performance in terms of print quality with the B9Creator. For repeatable and reliable printing, the resin viscosity under ambient conditions (25 °C) should remain below 0.2 Pas for the given setup without the use of an active surface scraper. The shear rate and temperature-dependent viscosity of the BAEDA–EGDMA resins were measured by cone-and-plate rheometry and are shown in Figure 7. The notation describes the by-wt.%-ratio of the two monomers, i.e., BE-5050 means 50 wt.% BAEDA and 50 wt.% EGDMA. The two monomers individually exhibit a simple Newtonian flow profile (Figure 7a), with the expected decrease in viscosities with increasing temperature (Figure 7b), in correlation with the Andrade equation [38].

## 5. Single-Material Print Mode Using the Base Resin BE-5050

**Figure 8:**
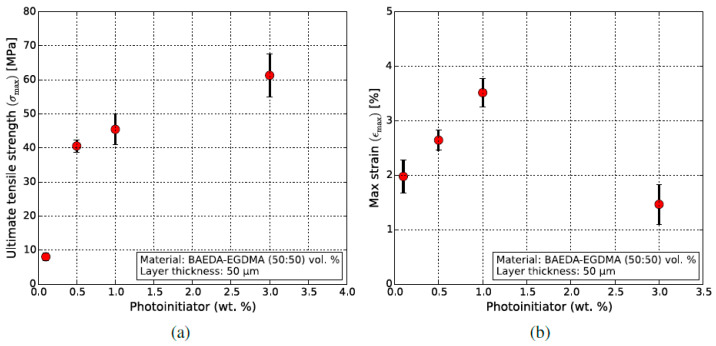
Impact of the photoinitiator content on the mechanical properties in BE-5050 base resin. (**a**) Ultimate tensile strength UTS; (**b**) Maximum strain at break.

Prior to its application in the MMSL, the B9Creator was used to validate the printability of BE-5050, which was selected as the base resin due to its good curing behavior, even with the presence of oxygen. To optimize the photopolymerization behavior, the mechanical properties of printed tensile test specimens (five samples per material composition) were measured as a function of photoinitiator content, which was set to between 0.1 wt.% and 3.0 wt.% (Figure 8). Increasing TPO contents force up the ultimate tensile strength (UTS) significantly (Figure 8a), whilst the maximum failure-strain has its maximum at 1.0 wt.%. TPO. Consequently, the TPO concentration was set to 1 wt.% for further experiments. Both of the used monomers possess two curable functional groups, hence after curing, a thermoset is synthesized. Increasing the photoinitiator content causes a rapid formation of a highly crosslinked system with increasing ultimate tensile strength and a reduced maximum strain.

**Figure 9:**
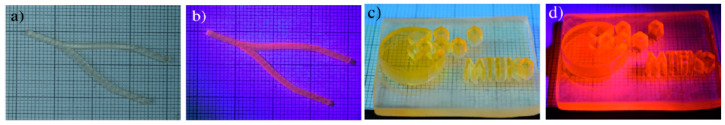
Single-material print with fluorescent dye-doped BE-5050. Printed with B9Creator: (**a**) Under ambient light; (**b**) Under a near UV LED. Printed with the MMSL device: (**c**) Under ambient light; (**d**) Under a near UV LED.

The usage of fluorescent dyes, dissolved in the base resin, helps to visualize the normally colorless and transparent printed parts. The demonstrators shown in Figure 9 were printed using the BE-5050 resin containing 5 wt.% of the fluorescent europium (III) complex. The B9Creator was used to print the Y-bend (Figure 9a,b), adapted from an optical application (beam splitter), at a resolution of 50 × 50 × 50 μm^3^ to investigate the polymerizability of the fluorescent resin. Figure 9c,d show the logo of the microsystem engineering department at University of Freiburg, printed by the MMSL device. Due to the absorption characteristics of the material, longer exposure times (30 s per layer, compared to 3 to 5 s for the unfilled resins) were required for sufficient polymerization. Using the MMSL device for the first time in the single-material mode at a voxel size of 30 × 30 × 30 μm^3^, the per-layer exposure time was reduced to 12 s due to the close proximity of the projector to the vat. Figure 9a,c show the test structures under ambient light and Figure 9b,d under near UV-light.

## 6. Dual Material Print Mode Applying the MMSL Device

### 6.1. Feasibility Study

**Figure 10:**
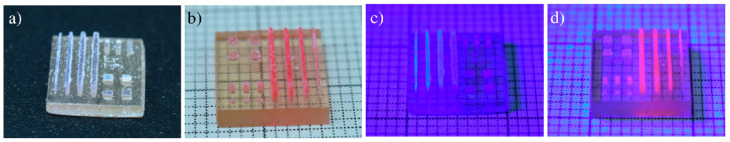
Microstructure demonstrators printed with the MMSL device, using a BE-8020 substrate with BE-5050 containing a fluorescent composite of the Lumogen V570 (**a,c**) and Lumogen F305 dyes (**b,d**); (**a**,**b**) Under ambient light; (**c**,**d**) Under near UV light.

The first feasibility studies of the new MMSL device used, as described in the previous section, fluorescent dyes as markers for the different applied resins. Figure 10 shows test patterns on a base plate. The base plate was made from pure BE-5050 and the test patterns used the same resin doped with the fluorescent dyes Lumogen V705 (Figure 10a,c) and F305 (Figure 10b,d). Especially under near UV-radiation, it is clearly visible that only the test patterns on top of the base plate were doped with the fluorescent dye. The MMSL device was shown to work reliably with two different materials at a minimum lateral resolution of 30 × 30 μm^2^, with freestanding structures down to 400 × 400 μm^2^ with an aspect ratio of 1 to 5.

### 6.2. Systematic Investigations

**Figure 11:**
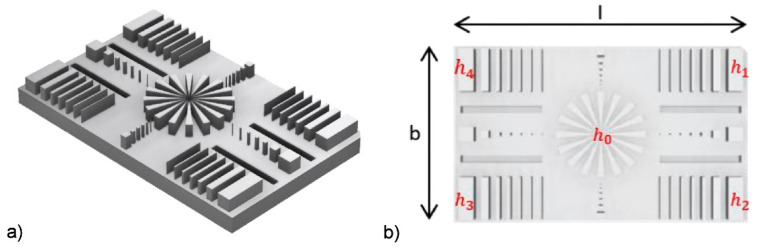
Benchmark artifact for the validation of the MMSL print quality; (**a**) Rendered 3D overview; (**b**) 2D projection with measuring points for structural feature heights.

To systematically investigate the print parameters, precision and accuracy, a benchmark artifact for the evaluation of the achievable MMSL geometric and structural accuracy performance was designed, following recommendations from the literature (Figure 11) [39,40]. The shown benchmark artifact contains structural features with a height of 2.5 mm on a 40 × 2 × 25 mm^3^ base plate. The geometric features of the cuboids range from 100 µm up to 2 mm, the columns have sizes from 100 × 100 µm^2^ up to 2 × 2 mm^2^ and the trenches have a depth of 100 µm. The central star-like structure possesses an outer diameter of 12 mm and an inner diameter of 1 mm. The small letters in Figure 11b describes the measuring points for sample height measurements.

#### 6.2.1. Single-Material Print

With ensure a reproducible printing quality, the printing behavior of the three different above-mentioned resins (BE-5050, BE-5050 doped with Lumogen F305 or Lumogen V570) were investigated systematically. The layer thickness was set to 50 µm; the curing time was varied between 1–4 s. All samples were post-processed after printing according to the listed parameters (Table 1).

**Figure 12:**
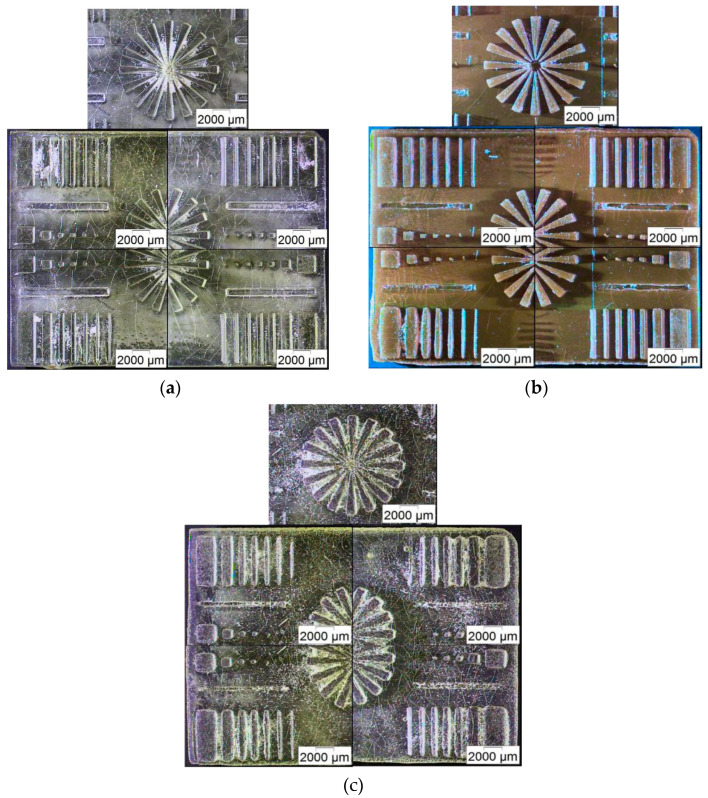
Microscopic images of the test patterns’ best layer curing time: (**a**) BE-5050; (**b**) BE-5050 Lumogen F305; (**c**) BE-5050 Lumogen V570.

For the different compositions and the respective absorption behaviors of the dopants, a slight difference in the best layer curing time was found. The visual appearance and the edge sharpness of the printed structures was selected as the main quality criterion. In the case of BE-5050, the best structure quality could be achieved with a layer curing time of 2 s, in which case only the smallest 100 × 100 µm^2^ columns were not repeatably producible. The curing defects in the middle of the star-like structure can be attributed to an insufficient monomer removal prior to the final flood illumination (Figure 12a). For BE-5050 doped with F305, a slightly longer curing time of 2.75 s was sufficient to form sharp edges (Figure 12b).

As in the pure base resin BE-5050, the smallest columns showed poor structure quality. The addition of Lumogen V570 caused a significantly reduced print quality (Figure 12c), where all edges and corners were rounded, and polymeric residue could be found between the individual structures, even at the best curing time of 2 s, and feature sizes below 200 × 200 µm^2^ could not be realized. The non-unique curing times can be attributed to the addition of the fluorescent dyes with their additional light absorption in the visible and near UV-range. When compared to the measured geometric features at the best curing layer time, all results remain close to the theoretical value (x-, y-directions) or stay constant (density), with exception of the average structure height, which differs significantly from the theoretical value of 4.5 mm (Table 2). This may be attributed to the poor spindle positioning of the z-axis and its reduced positional accuracy, originating from the spindle drive taken from the Makerbot Replicator 2X. Beyond the described feature properties, it is noticeable that the substrates often show surface defects. Micro-cracks within SLA-printed specimens arise from internal tensions during the polymerization process. These can be optimized for each resin variant by varying the light intensity and exposure time during printing, as well as during the post-print flood illumination. The systematic optimization of each resin variant (with or without a fluorescent dye), in terms of print parameters, lies outside the scope of this manuscript, which is a proof-of-concept for multi-material printing. The same is valid for the usage of commercial resins.

**Figure 13:**
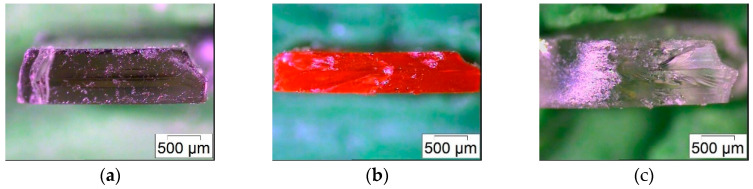
Fracture images after tensile testing. (**a**) BE-5050; (**b**) BE-5050\F305; (**b**) BE-5050\V570.

To overcome the subjective evaluation of the structural appearance, mechanical testing should deliver reliable data for the selected best layer curing time. Table 3 lists the resulting Young’s modulus and maximum tensile strength for the investigated curing times. Despite the significant experimental error, the curing time with the best structure quality delivers the highest maximum tensile strength as well. In general, an increase in the curing time should enhance the mechanical properties, for instance, due to a better intermixture accompanied with an enhanced polymer chain entanglement. Unfortunately, a clear correlation between curing time and mechanical properties cannot be found here, which may be attributed to the layer-by-layer fabrication process generating defects, as is prominently known for FFF printing [31]. A closer look at the fracture images after tensile testing verifies this possible explanation for the observed data scattering (Figure 13). A lamellar arrangement can be seen, in particular for the BE-5050 and BE5050\V750 systems, which reduces the mechanical stability due the presence of voids.

#### 6.2.2. Multi-Material Print

The same artifact structure, as shown in Figure 11, is taken for the evaluation of the multi-material print. The following steps describe the multi-material printing sequence:

(a) Material positioning: The two outer vats (Figure 4) are filled with the two materials to be printed (base plate resin and feature resin), while the middle vat is filled with isopropanol for rinsing.
(b) The material intended to be the base plate is printed using the steps described for single-material printing.
(c) The base plate is rinsed in isopropanol before moving to the vat with the feature material.
(d) The features are printed using single-material print parameters.
(e) The multi-material print is delaminated from the build-platform and post-processed, as described in Table 1.

**Figure 14:**
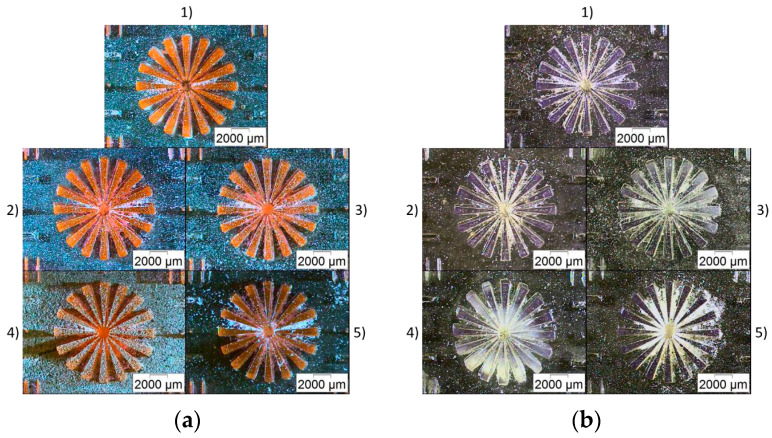
Microscopic images of the MMSL print applying BE-5050 as base plate material (five samples): (**a**) BE-5050\F305 as feature material; (**b**) BE-5050\V570 as feature material.

It has to be accentuated that only two different printing materials have been used, one for the base plate and a second one for the structural features. For the first attempt, BE-5050 was selected as the base plate material, in combination with the two doped systems. In all cases, five identically processed samples were printed, validating the printing reproducibility. Figure 14 shows the central star-like feature of five identical samples of BE-5050 – BE-5050\F305 (Figure 14a) and BE-5050 – BE5050\V570 (Figure 14b), demonstrating the reproducibility. Apart from a little polymerized residue in between the structures on the bottom, which may attributed to poor monomer removal during rinsing and cleaning, microstructured details of 250–300 µm (BE-5050 – BE-5050\F305) and 250–400 µm (BE-5050 – BE-5050\V570) could be realized in a repeatable manner. Both combinations possess a smooth surface and sharp edges. Dimensional measurements on the printed features and specimen properties showed (Table 4) similar results for both material combinations. As for single-material printing, the accuracy of the sample was fine, with the exception of the total structure height, which should be attributed to construction and calibration issues, especially to the positions of the different vats on the platform relative to each other. In all cases, a good adhesion of the second component on the first one was observed.

**Figure 15:**
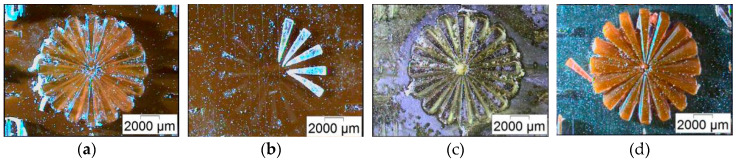
Representative microscopic images of different material combinations: (**a**) BE-5050\F305 as base plate with BE-5050 on top; (**b**) BE-5050\F305 as base plate with BE-5050\V570 on top; (**c**) BE-5050\V570 as base plate with BE-5050 on top; (**d**) BE-5050\V570 as base plate with BE-5050\F305 on top.

The artifact was additionally printed in an inverted arrangement, i.e., with one of the doped resins as the base plate and the other doped resin, or pure BE-5050, as the feature material. These showed worse print quality, as shown in Figure 15. With BE-5050\F305 as the base plate, the use of pure BE-5050 led to large monomer residue between the structures (Figure 15a). A very poor adhesion of BE-5050\V570 on the base plate can be seen, if used as the second system (Figure 15b), although the quality of the structures is better. The use of BE-5050\V570 as the substrate delivered good, reliable structural features and sharp edges (Figure 15c,d) and only small adhesion problems were observed. A closer look at the accuracy of the printed structures once again showed a good agreement with the x-, y-dimensions, and a significantly larger deviation of the nominal structure height (Table 5), which should be attributed to inaccuracies of the vat positioning in the z-direction. The smallest listed accessible structural feature was derived from the visual inspection of the structures regarding surface quality, edge sharpness and completeness.

#### 6.2.3. Best Accessible Resolution and Smallest Feature Size

**Figure 16:**
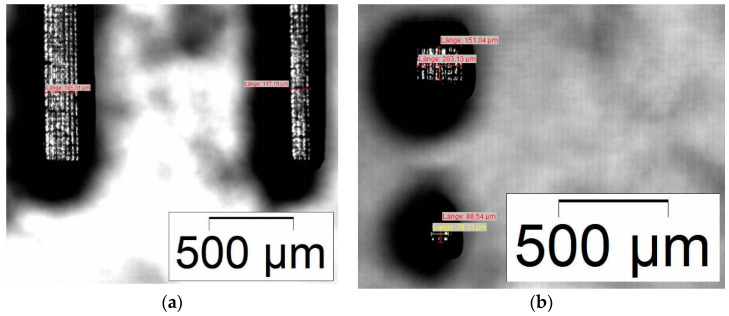
Single-material print: Microscopic images of printed micro-features with BE-5050\F305 resin; (**a**) columns; (**b**) cuboids.

**Figure 17:**
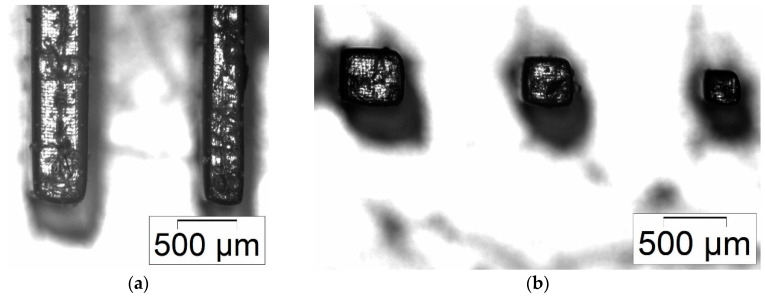
Multi-material print: Microscopic images of printed micro-features with BE-5050 as base plate material and BE-5050\F305 as feature material; (**a**) columns; (**b**) cuboids.

Whilst Figure 12, Figure 13, Figure 14 and Figure 15 give an overview about the general printability and reproducibility of single- and multi-material prints, a more detailed view is necessary to evaluate the structural quality and accessible print resolution. Figure 16 shows the surface quality of printed columns and cuboids for the single-material print (BE-5050\F305). The smallest measured lateral (xy) features are around 120 µm (Figure 16a) and around 90 µm (Figure 16b). Unfortunately, it was not possible to print these features at this size in a reproducible manner, hence the smallest reproducible measured lateral (xy) features were around 200 µm. In all cases, a good retention of the edges can be observed, at the top face, a grid-like pattern originating from the DMD mirrors of the DLP projector can be seen. Concerning multi-material printing, Figure 17 shows the surface quality of printed columns and cuboids for the combination of BE-5050 as the base and BE-5050\F305 as the feature material. The smallest measured features are around 250 µm.

### 6.3. Process Evaluation

The aforementioned results show that the new MMSL equipment is able to combine two different curable resins in an acceptable manner. With respect to accuracy and reproducibility, two points have to be considered. The data shown in Table 2, Table 4 and Table 5, for the geometric features and densities, are the mean values over five samples printed under identical conditions. The standard deviation for these results is acceptable. The reproducibility of the feature quality as a function of the printing parameters is not unique. In the case of the single-material printing surface quality, the presence of defects and rounded edges varies within one set of samples printed using identical parameters, especially in the case of the doped systems BE-5050\F305 and BE-5050\V570. With respect to accuracy and precision, especially in the case of structural details significantly below 500 µm, some construction-related improvements can be made. Firstly, the spindle used for the z-direction control has to be substituted by a more precise one, i.e., one with a lower pitch, so that the stepper motor can deliver the desired positional accuracy (see Section 3.2). Secondly, the mounting of the vats on the build-platform, and the build-platform calibration relative to the vat window, should be improved, enabling plane–parallel operation and a more precise deposition of the second material on the first one. The majority of observed print defects can be traced back to misalignment issues.

## 7. Conclusions and Outlook

Within this work, a comprehensive approach for the realization of a low-cost multi-material stereolithography 3D printing device was presented, ranging from construction, software control, material development and characterization, as well as the first printing results with two different curable resins with similar chemistry. It was possible to print two-component test parts with the smallest structural features in the 200–300 µm range. The feasibility of low-cost multi-material printing using parts from defunct machines and inexpensive components was successfully demonstrated. If one wants to realize smaller structures in a better quality, accuracy and printing reliability, a significant process improvement is necessary. This can be achieved, e.g., by the assembly of high performance positioning elements. Looking ahead, the MMSL printer is a versatile device capable of being tuned to work with other resin systems and fabricating structures with enhanced mechanical and functional properties.

## Figures and Tables

**Table 1:** Post processing parameters after MMSL printing.

Step	Parameter
Rinsing in ultrasonic bath	5 min, isopropanol
Flood illumination wavelength	385 nm
Flood illumination intensity	200 mW/cm^2^
Flood illumination time	100 s
Flood illumination atmosphere	N_2_

**Table 2:** Sample features at best layer curing time estimated by visual inspection (mean values).

Item	BE-5050	BE-5050\F305	BE-5050\V570
Polymerization time per layer (s)	2	2.75	2
Height (mm)	3.67 ± 0.20	2.92 ± 0.28	3.80 ± 0.31
Density (g/cm^3^)	1.21 ± 0.00	1.22 ± 0.01	1.21 ± 0.00
Length (mm)	38.90	39.14	39.35
Width (mm)	24.52	25.04	24.93
Smallest structural detail (µm)	100–200	100–200	400

**Table 3:** Mechanical properties of all investigated systems as function of the layer curing time.

Item	BE-5050			BE-5050\F305			BE-5050\V570		
Curing time (s)	1	2	3	2	2.75	3.5	1	2	2.5
Young’s module (MPa)	236 ± 124	474 ± 78	459 ± 53	614 ± 193	578 ± 152	781 ± 144	261 ± 113	348 ± 61	304 ± 51
Max. tensile strength (MPa)	16 ± 4	30 ± 11	30 ± 5	26 ± 13	40 ± 12	35 ± 14	26 ± 5	36 ± 7	24 ± 8

**Table 4:** Sample features of MMSL print with BE-5050 as base plate in combination with BE-5050\F305 or BE-5050\V570 (average values).

Item	BE-5050\F305	BE-5050\V570
Height (mm)	3.42 ± 0.44	3.20 ± 0.56
Density (g/cm^3^)	1.20 ± 0.00	1.20 ± 0.01
Length (mm)	39.39 ± 0.17	39.26 ± 0.29
Width (mm)	24.91 ± 0.07	24.81 ± 0.04
Smallest structural detail (µm)	250	250

**Table 5:** Sample features of MMSL print with different material combinations (average values).

Item	Base:Top:	BE-5050\F305 BE-5050	BE-5050\F305 BE-5050\V570	BE-5050\V570 BE-5050	BE-5050\V570 BE-5050\F305
Height (mm)	-	2.84 ± 0.42	2.10 ± 0.24	3.56 ± 0.17	1.89 ± 0.52
Length (mm)	-	39.13 ± 0.40	39.50 ± 0.26	39.11 ± 0.10	39.76 ± 0.30
Width (mm)	-	25.02 ± 0.42	25.13 ± 0.12	24.96 ± 0.29	25.47 ± 0.11
Smallest structural detail (µm)	-	300	300	200	200
